# Ovarian volume and antral follicle count for the prediction of low and hyper responders with in vitro fertilization

**DOI:** 10.1186/1477-7827-5-9

**Published:** 2007-03-15

**Authors:** Janet Kwee, Mariet E Elting, Roel Schats, Joseph McDonnell, Cornelis B Lambalk

**Affiliations:** 1 Vrije Universiteit Medical Center, PO Box 7057, 1007 MB Amsterdam, the Netherlands; 2 Division of Reproductive Endocrinology and Fertility and the IVF Centre, Department of Obstetrics and Gynaecology, Vrije Universiteit Medical Centre, Amsterdam, the Netherlands; 3 Department of Clinical Genetics, Vrije Universiteit Medical Centre, Amsterdam, the Netherlands

## Abstract

**Background:**

The current study was designed to compare antral follicle count (AFC) and basal ovarian volume (BOV), the exogenous FSH ovarian reserve test (EFORT) and the clomiphene citrate challenge test (CCCT), with respect to their ability to predict poor and hyper responders.

**Methods:**

One hundred and ten regularly menstruating patients, aged 18–39 years, participated in this prospective study, randomized, by a computer designed 4-blocks system study into two groups. Fifty six patients underwent a CCCT, and 54 patients underwent an EFORT. All patients underwent a transvaginal sonography to measure the basal ovarian volume and count of basal antral follicle. In all patients, the test was followed by a standard IVF treatment. The result of ovarian hyperstimulation during IVF treatment, expressed by the total number of follicles, was used as gold standard.

**Results:**

The best prediction of ovarian reserve (Y) was seen in a multiple regression prediction model that included, AFC, Inhibin B-increment in the EFORT and BOV simultaneously (Y = -3.161 + 0.805 × AFC (0.258-1.352) + 0.034 × Inh. B-incr. (0.007-0.601) + 0.511 BOV (0.480-0.974) (r = 0.848, p < 0.001). Univariate logistic regression showed that the best predictors for poor response were the CCCT (ROC-AUC = 0.87), the bFSH (ROC-AUC = 0.83) and the AFC (ROC-AUC = 0.83). Multiple logistic regression analysis did not produce a better model in terms of improving the prediction of poor response. For hyper response, univariate logistic regression showed that the best predictors were AFC (ROC-AUC = 0.92) and the inhibin B-increment in the EFORT (ROC-AUC = 0.92), but AFC had better test characteristics, namely a sensitivity of 82% and a specificity 89%. Multiple logistic regression analysis did not produce a better model in terms of predicting hyper response.

**Conclusion:**

In conclusion AFC performs well as a test for ovarian response being superior or at least similar to complex expensive and time consuming endocrine tests. It is therefore likely to be the test for general practise.

## Background

Real time two-dimensional (2D) pelvic ultrasonography is a relatively accurate and reliable method of determining ovarian volume and morphology [1]. Interobserver and intraobserver measurements have been shown to be very low when using transvaginal sonography [2,3].

The mean ovarian volume increases from 0.7 ml at 10 years to 5.8 ml at 17 years of age [4]. It has been suggested that there are no major changes in ovarian volume during reproductive years until the premenopausal period. In women > 40 years old, there is a dramatic drop in ovarian volume, which is not related to parity [2,4,5]. Thereafter there is a further sharp decline in size in postmenopausal women which seems mostly related to the time when menstruation ceases, rather than merely to age, because when oestrogen treatments were given, there appeared to be no decrease in ovarian volume with age [5].

Several studies [6-8] demonstrate that ovarian volume, as determined by transvaginal ultrasonography, is a predictor of ovarian reserve and clinical pregnancy rate. Lass *et al*. [9] confirmed that decrease in ovarian volume is an early sign of depletion of the follicles and its measurement is likely to be clinically useful.

A cohort of follicles measuring 2–5 mm is present very early in the follicular phase of the cycle [10]. These follicles are in an early antral phase, and are easily detected by transvaginal ultrasound, as they contain a small amount of antral fluid. The number of small follicles at the beginning of the cycle may well represent the actual functional ovarian reserve. So the number of small antral follicles are clearly related to age and could well reflect the size of the remaining primordial pool in women with proven natural fertility [11,12].

Previously [13], we published the comparison of endocrine tests for the prediction of the total number of follicles obtained after stimulation. With linear regression analysis, Inhibin B-increment and E2-increment in the EFORT gave the best predictive values. We tried to find one single, simple test, which could identify poor, normal and hyper responders [14] and concluded that by logistic regression analysis, the bFSH + sFSH in the CCCT was the best endocrine test to predict poor responders, unfortunately not for the prediction of hyper responders. The aim of the current study was to compare the antral follicle count (AFC) and the basal ovarian volume (BOV), with the exogenous FSH ovarian reserve test (EFORT) and the clomiphene citrate challenge test (CCCT), with respect to their ability to predict poor and hyper responders.

## Methods

### Study population

One hundred and ten patients, aged 18–39 years, who were eligible for treatment by Intra Uterine Insemination (IUI) between June 1997, to December 1999, participated in the study. This study is part of a prospective randomized study of regular menstruating patients to the determination of ovarian reserve [13]. Their infertility was either idiopathic for > 3 years and/or due to a male factor and/or cervical hostility. Cervical hostility was diagnosed by means of a well timed negative postcoital test, that is, no progressive motile spermatozoa seen at a magnification of 400× in good cervical mucus despite normal semen parameters.

Patients had to have regular menstrual cycles with an ovulation, which was confirmed by a biphasic body temperature chart and an endometrium biopty dating in the luteal phase, two ovaries and showed two patent tubes with hysterosalpingography or at least one patent Fallopian tube with no further pathology with diagnostic laparoscopy. They were naive for IVF treatment. Excluded were patients with an oligo- or amenorrhoea (9 or fewer cycles a year) or a severe male factor, defined as (1) less than 1 million motile spermatozoa after Percoll centrifugation (gradient 40/90) and/or (2) > 20% antibodies present on the spermatozoa after processing with Percoll centrifugation (gradient 40/90) and/or (3) > 50% of the spermatozoa without an acrosome. Other exclusion criteria were untreated or insufficiently corrected endocrinopathies, clinically relevant systemic diseases, or a body mass index > 28 kg/m^2^.

The protocol was approved by the Institutional Review Board and the Committee on ethics of research involving human subjects of the Vrije Universiteit Medical Centre, Amsterdam, the Netherlands. All the couples participating in the study signed informed consent.

### Treatment protocol

Patients were randomized by a computer designed 4-blocks system into two groups [13]. Fifty six patients underwent a transvaginal sonography to measure the basal ovarian volume and count of basal antral follicle and a Clomiphene citrate challenge test (CCCT), and 54 patients underwent an transvaginal sonography to measure the basal ovarian volume and count of basal antral follicle and an Exogenous Follicle stimulating hormone Ovarian Reserve Test (EFORT). In all patients, the test was followed by an IVF treatment under a long protocol. The bFSH level, bE2 level and bInhibin B level were determined as an integral part of all CCCT's and EFORT's, as described previously [13]. Van der Meer *et al*. [15] showed that in eumenorrheic patients, the median (range) FSH threshold level for monofollicular growth was 5.3 (4.3–8.2) IU/l and the median (range) threshold dose was 75 IU (0.5–1.75) FSH/day.

The FSH threshold was determined by a low dose step-up regimen of FSH given intravenously after pituitary desensitization with GnRH agonist. It was concluded that by an increment of 1/2 ampoule of FSH (37.5 IU) above the threshold dose for monofollicular growth, the maximum response is already obtained. It seems that in IVF stimulation maximal effect is reached with FSH dosages up to 225 IE [16-18]. Combining these facts, it can be concluded that an initial stimulation by 3 ampoules of 75 IU of FSH under a long (GnRH agonist suppressed) protocol, probably gives a maximal IVF stimulation, the outcome of which could be used as the gold standard for the cohort size.

#### Transvaginal sonography measurements

All ultrasound examinations were performed by one of the authors (J.K, R.S) using an Aloka SSD-1700 ultrasound apparatus (5.0 MHz probe).

The volume of each ovary was calculated by measuring in three perpendicular directions and applying the formula for an ellipsoid: (D1 × D2 × D3 × π/6). The volumes of both ovaries were added for the total basal ovarian volume (BOV).

To determine the diameter of the follicle, the mean of measurements in two perpendicular directions was taken. The numbers of follicles in both ovaries were added for the total antral follicle count (AFC). The follicles visualized and counted by TVS in the early follicular phase are 2–10 mm in size.

#### Clomiphene citrate challenge test (CCCT)

starting on the fifth day of the menstrual cycle (CD 1 = day of onset of menses) 100 mg of Clomiphene citrate (Serophene^®^; Serono, Geneva, Switzerland) was administered for 5 days. In this study on CD 2 or 3 (basal values) and on CD 10 (stimulated values) the serum FSH was determined. Analysis of the CCCT [13] was performed by the parameter: bFSH + sFSH.

#### Exogenous Follicle stimulating hormone Ovarian Reserve Test (EFORT)

on CD 3, 300 IU recFSH (Gonal-F^®^, Serono, Geneva, Switzerland) were administered subcutaneously (s.c). In this study blood samples for the determination of FSH, E2 and Inhibin B were drawn: just before (basal values) and 24 hrs after (stimulated values) the administration of FSH. Analysis of the EFORT [13] included the following parameters: E2-increment and Inhibin B-increment 24 hrs after administration of FSH.

#### IVF-treatment

The ovarian hyperstimulation protocol was performed according to a long GnRH-agonist protocol starting in the midluteal phase. On CD 3 of the first cycle the ovarian volume and antral follicle count was measured by transvaginal sonography (TVS) examinations as described above. Also on CD 3 the CCCT or the EFORT was performed as described above. In the subsequent midluteal phase, seven days after ovulation, daily s.c. injections with triptoreline-acetate (Decapeptyl^®^, 0.1 mg/day; Ferring, Hoofddorp, the Netherlands) were started. Because of the administration of the GnRH-agonist, patients were advised to use a barrier type of contraception during this cycle. On CD 3 of the next cycle, ovarian hyperstimulation was started with daily s.c. injections of a fixed dose of 225 IU uFSH (Metrodin HP^®^, 75 IU/amp; Serono, Geneva, Switzerland), because this dosage probably gives a maximal effect in follicle stimulation. Standard procedures were followed including transvaginal sonography (TVS) (Aloka SSD-1700, 5.0 MHz probe) on CD 2 or 3 and on CD 9 or 10. Daily TVS was performed from the moment when the leading follicle reached a diameter of 16 mm. Ovarian hyperstimulation was continued until the largest follicle reached a diameter of at least 18 mm. The maximum duration of uFSH administration allowed was 16 days. If these criteria were met, Metrodin HP^® ^and Decapeptyl^® ^were discontinued and 10.000 IU of hCG (Profasi^®^, 10.000 IU/amp; Serono, Geneva, Switzerland) were administered. On the day of hCG, TVS was performed to count the result of ovarian hyperstimulation (all follicles = 10 mm) expressed as the total number of follicles. TVS guided follicular aspiration (FA) was performed 36 hours after hCG administration. On the day of hCG administration E2 was determined. Follicular aspiration was done under systemic analgesia (7.5 mg diazepam orally and 50–100 mg pethidine hydrochloride intramuscularly), and all follicles present were aspirated.

### Serum assay

Serum E2 was determined by a competitive imunoassay (Amerlite, Amersham, UK). For E2, the inter-assay CV was 11% at 250 pmol/l and 8% at 8000 pmol/l, the intra-assay coefficient of variation (CV) was 10% at 350 pmol/l. 8% at 1100 pmol/l and 8% at 5000 pmol/l. The lower limit of detection for E2 was 90 pmol/l. In the EFORT and CCCT we measured E2 by a sensitive radioimmunoassay (Sorin, Biomedica, Saluggia, Italy). This measurement of E2 was abbreviated as EE. For EE, the inter-assay CV was 11% at 60 pmol/l, 8% at 200 pmol/l, 11% at 550 pmol/l and 8% at 900 pmol/l. The intra-assay CV was 4% at 110 pmol/l and 5% at 1000 pmol/l. The lower limit of detection for EE was 18 pmol/l. FSH was determined by a commercially available immunometric assay (Amerlite, Amersham, UK). For FSH, the inter-assay CV was 9% at 3 IU/l and 5% at 35 IU/l, the intra-assay CV was 9% at 5 IU/l, 8% at 15 IU/l and 6% at 40 IU/l. The lower limit of detection for FSH was 0.5 IU/l. Inhibin B was determined immunometrically by a commercially available assay (Serotec Limited Oxford UK). For Inhibin B, the inter-assay CV was 17% at 25 ng/L, 14% at 55 ng/L and 9% at 120 ng/L and the intra-assay CV was 8% till 40 ng/l and 5% at > 40 ng/l. The lower limit of detection for Inhibin B was 13 ng/l.

Half-way through the study (after 62 patients), the Amerlite assay used to assess FSH was withdrawn from the market and was replaced by another commercially available assay (Delfia, Wallac, Finland). The two assays have been compared and showed excellent linear correlation, although a shift in the values took place (Delfia FSH = 1.28 × Amerlite FSH + 0.01 (r = 0.9964)). For the Delfia FSH, the inter-assay CV was 5% at 3.5 IU/l and 3% at 15 IU/l. All FSH determinations have been recalculated and are expressed according to the Delfia assay. The lower limit of detection for FSH was 0.5 IU/l.

Values below the detection limit of an assay were assigned a value equal to the detection limit of that assay.

### Statistical analysis

The outcome measure of the first part of this study was the result of ovarian hyperstimulation expressed as the number of follicles. In our former study [13], we estimated the value of the independent variables by univariate linear regression, age, bFSH, CCCT-results, E2-increment in EFORT, inhibin B-increment in EFORT. In this study, we estimated by univariate linear regression, the value of the independent variables: total basal ovarian volume and the total basal antral follicle count in predicting the ovarian response. Stepwise regression analysis was used to find a prediction model for the ovarian response. The R square of the correlation of these variable(s) with the total number of follicles obtained after stimulation reflects the proportion of the variability of the number of follicles explained by this variable(s).

The outcome measure of the second part of this study was the result of ovarian hyperstimulation expressed as the number of retrieved oocytes.

We defined a 'poor' ovarian response as less than 6 oocytes after ovarian hyperstimulation in an IVF treatment and a 'hyper' response as more than 20 oocytes after such an IVF treatment. Among women undergoing in vitro fertilization, the chances of a live birth are related to the number of eggs fertilized, presumably because of the greater selection of embryos for transfer. The low success rate when only two eggs were fertilized reflects the lack of choice among embryos for transfer [19]. We have in our laboratory the experience that we have an overall 50–60% chance of fertilisation. Taken this togheter, at least 6 oocytes are required for three or more fertilized eggs.

We defined a hyper response when there were > 20 oocytes. This was based on the knowledge that the pregnancy rates do not increase when > 20 oocytes are retrieved. Moreover, such cases have a significant risk of a severe OHSS [14].

In our former study [14], we examined the value of the independent variables by univariate logistic regression: age, bFSH, binhibin B, CCCT-results, E2-increment in EFORT, inhibin B-increment in EFORT. In this study we examined by univariate logistic regression, the value of the independent variables: total basal ovarian volume and the total basal antral follicle count in predicting the ovarian response in predicting a poor and hyper response after ovarian hyperstimulation in IVF. Subsequently multivariate logistic regression analyses were used to develop prediction models for the ovarian response. The area under the receiver operating characteristic curve (ROC-AUC) was computed to assess the predictive accuracy of the logistic models. ORT evaluation using ovarian response as reference or outcome variable should imply the assessment of predictive accuracy and clinical value of the test. Accuracy refers to the degree by which the outcome condition is predicted correctly. Summary statistics of accuracy include *sensitivity *(rate of correct identification of cases with poor response) and *specificity *(rate of correct identification of cases without poor response). To identify all cases that will respond poorly to stimulation without judging many normal responders badly, the test must have high sensitivity and high specificity.

The Receiver Operating Characteristic curve (ROC curve) is a plot of the true positive rate against the false positive rate for the different possible cutpoints of a diagnostic test. An ROC curve demonstrates the tradeoff between sensitivity and specificity (any increase in sensitivity will be accompanied by a decrease in specificity). The closer the curve follows the left-hand border and then the top border of the ROC space, the more accurate the test. The *area under the ROC curve *provides information on the overall discriminatory capacity of the test. Values of 1.0 imply perfect and that of 0.5 indicate completely absent discrimination.

To define a 'normal' and an 'abnormal' test, sensitivity, specificity, positive predictive value and accuracy were used to find the optimal cut off level.

Comparison of means was done with the unpaired t-test. For all tests the significance level was 0.05.

Statistical analysis of the data was performed with SPSS (Statistical package for Social Sciences; SPSS, Inc., Chicago, IL) for Windows.

## Results

The characteristics of the two groups are given as means ± SD in Table 1[13]. No significant differences were noted between the groups in baseline characteristics, cycle day 3 measurements or outcome parameters. In the first group 68% had a primary infertility and 32% a secondary infertility. The cause of infertility was for 65% an idiopathic factor, 31% a male factor and 4% a cervical factor. In the second group, 60% had a primary infertility and 40% a secondary infertility. The cause of infertility was for 66% an idiopathic factor, 38% a male factor and 6% a cervical factor.

**Table 1:** Characteristics of the groups (values are means ± SD). No significant differences

	CCT-group N = 56	EFORT-group N = 54
*Baseline characteristics*		
Age (y)	33.79 ± 3.95	34.19 ± 3.75
Duration infertility (y)	3.71 ± 2.08	3.87 ± 1,56
*Cycle day 3*		
FSH (IU/l)	7.60 ± 2.46	7.38 ± 3.11
E2 (pmol/l)	126.05 ± 53.10	118.60 ± 47.06
Inhibin B (ng/l)	94.95 ± 39.36	96.33 ± 40.60
Total volume (ml)	10.91 ± 5.19	12.10 ± 4.69
Total antral follicle count	9.41 ± 5.00	10.66 ± 5.21
*Treatment results*		
Duration of stimulation (d)	12.4 ± 2.7	11.9 ± 2.3
Number of ampoules of FSH	34.2 ± 8.0	32.7 ± 7.0
E2 level on the day of hCG (pmol/l)	11155.41 ± 18591.13	12134.78 ± 17872.12
*Endpoints*		
Total number of follicles	14.27 ± 10.23	14.17 ± 10.27
Total number of oocytes	11.58 ± 8.51	11.93 ± 9.11

In the CCCT group 32 patients had a normal response to ovarian stimulation, 15 patients had a poor response and 8 patients had a high response. One patient was excluded from analysis because of a severe risk on ovarian hyperstimulation syndrome (OHSS) (the E2 level exeeded 35.000 pmol/l after ovarian hyperstimulation). In the EFORT group 32 patients had a normal response to ovarian stimulation, 14 patients had a poor response and 8 patients had a high response.

### Univariate linear regression analysis

**Figure 1:**
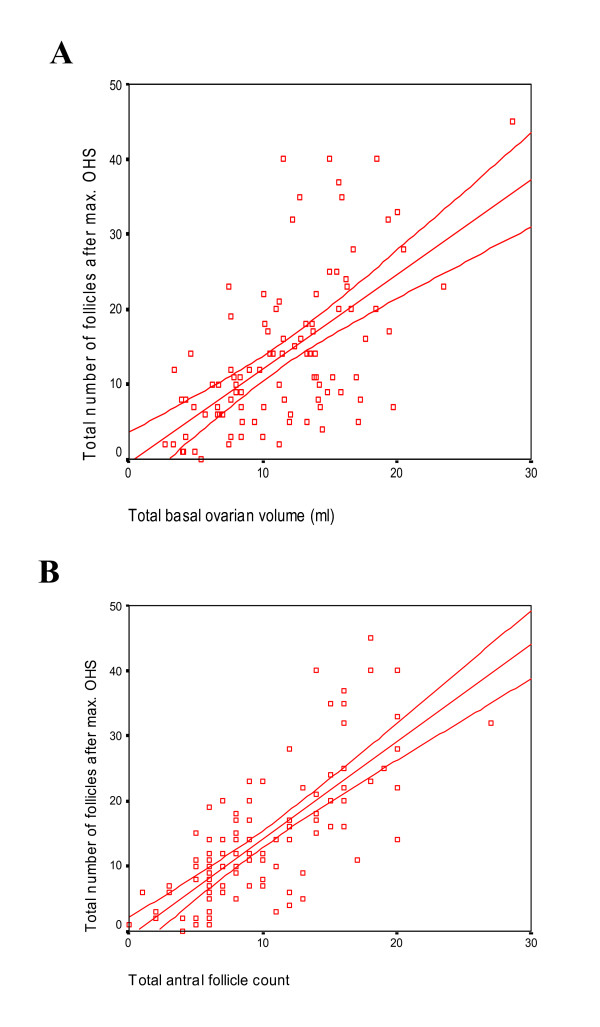
(**A**) Plot of the number of follicles obtained after stimulation against the basal total ovarian volume. The three lines represent the regression line: Y = -0.460 + 1.255 × tot. ovarian volume with the 95% confidence interval (CI) of the mean. (**B**) Plot of the number of follicles obtained after stimulation against the basal total antral follicle count. The three lines represent the regression line: Y = -0.730 + 1.491 × tot. antral follicle count with the 95% CI of the mean.

The correlation between the total basal ovarian volume (BOV) and number of follicles obtained after stimulation and the correlation between the count of the total basal antral follicle (AFC) and number of follicles obtained after stimulation are calculated. The regression line of the basal ovarian volume on the number of follicles (Figure 1A) was drawn by the regression equitation: X = -0.211 + 1.239 × tot. Volume; with a 95% CI of 0.909–1.569, meaning that each increment of 1 ml of ovarian volume predicts an increment of 1.2 follicle (95% CI: 0.9–1.6) (r = 0.610, P < 0.001). The regression equitation for the total basal antral follicle (Figure 1B), X = -0.568 + 1.479 × tot. antral foll. (1.222–1.736), shows that an increase of 1 antral follicle predicts an increment of 1.5 follicles (r = 0.741, P < 0.001).

Table 2 shows the results of the EFORT and CCCT as described in our previous study [13] and the additional results of the transvaginal ultrasound.

**Table 2:** Univariate regression analysis of the ovarian reserve tests for the prediction of the stimulative cohort of the ovaries (ovarian reserve).

	N	Correlation	P
Age (y)	110	0.423	< 0.001
bFSH (IU/l)	110	0.313	0.001
bFSH + sFSH in the CCCT (IU/l)	56	0.496	< 0.001
E2-increment in the EFORT (pmol/l)	54	0.751	< 0.001
Inh.B-increment in the EFORT (ng/l)	54	0.718	< 0.001
Total ovarian volume (ml)	110	0.610	< 0.001
Total antral follicle count	110	0.745	< 0.001

### Stepforward regression analysis: Prediction model for ovarian reserve

Based on the CCCT group, the prediction model for ovarian response is explained for 51% by the best predictive variable: the total antral follicle count. When adding the independent variables: total basal volume, bFSH + sFSH, bFSH and age in a stepforward regression analysis, the explained variation rose significantly with 5% after the selection of bFSH. The independent variable total basal volume, ∑ bFSH + sFSH and age, did not have a significant contribution to the model. The exact prediction of the total number of follicles obtained after stimulation thus increased from 51% to 56%. The regression line of the bFSH and total antral follicle count on the number of follicles was drawn by the regression equation: Y = 9.478 - 0.985 × bFSH (-1.857- -1.150) + 1.122 × AFC (0.698 -1.561) (r = 0.748, p < 0.001).

Based on the EFORT group, the prediction model for ovarian response is explained for 63% by the best predictive variable, the total antral follicle count. When adding the Inhibin B-increment and total basal volume simultaneously in a stepforward multiple regression prediction model, the explained variation of the best predictive model rose significantly with 9%. The total explained variation thus increased from 63% to 72%. The regression line of the total antral follicle count, Inhibin B-increment and total basal volume on the number of follicles was drawn by the regression equation: Y = -3.161 + 0.805 × AFC (0.258-1.352) + 0.034 × Inh. B-incr. (0.007-0.601) + 0.511 BOV (0.480-0.974) (r = 0.848, p < 0.001). When we included E2-increment, age and bFSH as variables in the stepforward regression analysis together with total antral follicle count, Inhibin B-increment and the total basal ovarian volume we did not find a significant contribution of these variables.

### Univariate logistic regression

Table 3 depicts the ROC-AUC for the total basal ovarian volume and the total basal antral follicle count for the prediction of poor response after IVF with ovarian hyperstimulation and also the results of the EFORT and CCCT as described previously [14]. Both tests have the potential to predict poor response, expressed by the ROC-AUC (0.83 respectively, 0.77).

**Table 3:** Univariate and multivariate logistic regression analysis and areas under the receiver operating characteristic curve (ROC-AUC) of the ovarian reserve tests for the prediction of 'poor' response in IVF.

Variable	N	P	ROC AUC
*Univariate analysis*			
Age (y)	110	0.033	0.63
bFSH (IU/l)	110	< 0.0001	0.83
bFSH + sFSH in the CCCT (IU/l)	56	< 0.0001	0.88
E2-increment in the EFORT (pmol/l)	54	0.006	0.75
Inh.B-increment in the EFORT (ng/l)	54	< 0.0001	0.86
Total ovarian volume (ml)	110	< 0.0001	0.77
Total antral follicle count	110	< 0.0001	0.83
*Multivariate analysis* CCCT GROUP			
bFSH + sFSH in the CCCT (IU/l))	56	< 0.0001	0.88
*Multivariate analysis* EFORT GROUP			
Total antral follicle count	54	0.003	0.88

Table 4 presents test characteristics such as sensitivity, specificity, positive predictive value and accuracy at different cut off levels of the AFC to define a normal (non-poor response) and an abnormal (poor response) test for the prediction of 'poor'response after IVF. The cut off level of < 6 antral follicles had a sensitivity of 41% and a specificity of 95%. In the population studied, with a prevalence of 27% for a poor response (< 6 oocytes after ovarian hyperstimulation in an IVF treatment), the accuracy was 89% (which means that 89% of the patients had a correctly predicted test). In case of a result less than 6 antral follicles, the test correctly predicted poor response to stimulation in an IVF-treatment in 75% (positive predictive value).

**Table 4:** Sensitivity, specificity, positive predictive value (PPV) for positive test results and proportion of patients (accuracy) with a correct prediction at different cut off levels for the total antral follicle count (AFC) for the prediction of 'poor'response in IVF.

Total AFC	Sensitivity	Specificity	PPV	Accuracy
< 4	0.21	0.99	0.86	0.78
< 5	0.28	0.99	0.89	0.80
**< 6**	**0.41**	**0.95**	**0.75**	**0.89**
< 7	0.69	0.80	0.56	0.77
< 8	0.76	0.74	0.51	0.75

Table 5 depicts ROC-AUC for the total basal ovarian volume and the total basal antral follicle count for the prediction of hyper response after IVF with ovarian hyperstimulation and also the results of the EFORT and CCCT as described previously [14]. As a single prognostic predictor, the AFC appeared to have a good discriminative potential for hyper response, expressed by a large ROC-AUC (0.92).

**Table 5:** Univariate and multivariate logistic regression analysis and areas under the receiver operating characteristic curve (ROC AUC) of the ovarian reserve tests for the prediction of hyper 'response' in IVF.

Variable	N	P	ROC AUC
*Univariate analysis*			
Age (y)	110	0.004	0.71
BFSH (IU/l)	110	< 0.0001	0.80
bFSH + sFSH in the CCCT (IU/l)	56	0.003	0.82
E2-increment in the EFORT (pmol/l)	54	0.003	0.83
Inh.B-increment in the EFORT (ng/l)	54	< 0.0001	0.92
Total ovarian volume (ml)	110	< 0.0001	0.87
Total antral follicle count	110	< 0.0001	0.92
*Multivariate analysis* CCCT GROUP			
Age	56	0.032	0.93
Total antral follicle count	56	<0.0001	
*Multivariate analysis* EFORT GROUP			
Total antral follicle count	54	<0.0001	0.93

Table 6 presents test characteristics such as sensitivity, specificity, positive predictive value and accuracy at different cut off levels of the AFC to define a normal (non-high response) and an abnormal (hyper response) test for the prediction of hyper response after IVF. The cut off level of > 14 antral follicles gave the highest sum of the sensitivity, specificity and gave also the highest accuracy. This result had a sensitivity of 82% and a specificity of 89%. In the population studied, with a prevalence of 15% for high response (> 20 oocytes after ovarian hyperstimulation in an IVF treatment), the accuracy was 88% (which means that 88% of the patients had a correct predicted test). In case of a result of greater than 14 antral follicles, the test correctly predicted hyper response to stimulation in an IVF-treatment in 58% (positive predictive value).

**Table 6:** Sensitivity, specificity, positive predictive value (PPV) for positive test results and proportion of patients (accuracy) with a correct prediction at different cut off levels for the total antral follicle count (AFC) for the prediction of 'hyper'response in IVF.

Total AFC	Sensitivity	Specificity	PPV	Accuracy
> 10	0.94	0.71	0.36	0.76
> 12	0.88	0.80	0.44	0.81
**> 14**	**0.82**	**0.89**	**0.58**	**0.88**
> 16	0.47	0.96	0.67	0.88
> 18	0.29	0.98	0.71	0.87

### Multivariate logistic regression

In the CCCT group, multivariate analysis for poor response resulted in a model with 1 variable: bFSH + sFSH in the CCCT (ROC-AUC = 0.88).

In the EFORT group, multivariate analysis for poor response resulted in a model with only one variable: Total antral follicle count (ROC-AUC = 0.88) (Table 3).

In the CCCT group, multivariate analysis for hyper response resulted in a model with 2 variables: age and AFC (ROC-AUC = 0.93).

In the EFORT group, multivariate analysis for hyper response resulted in a model with only one variable: AFC (ROC-AUC = 0.93) (Table 5).

## Discussion

AFC is able to accurately predict the number of follicles obtained during maximal ovarian stimulation. According to our study that uniquely allowed direct comparison, AFC does not seem superior to other common basal and stimulated endocrine ovarian reserve tests. Included into the stepwise forward multiple regression model it leads, in combination with the Inhibin B-increment in the EFORT and BOV, to the most optimal prediction model. On the other hand, according to logistic analysis, AFC sofar seems to be the only test able to reliably predict low and high responders.

Reproductive aging is thought to be dictated by a gradual decrease in both the quantity and the quality of the oocytes and follicles held within the ovaries [20,21]. With regard to quantity, histological studies have shown that at birth a few million primordial follicles are present from which at the onset of puberty only some 400.000 are left [22-25]. The wasting of follicles continuous throughout reproductive life, reaching a critical number of a few thousand at a mean age of 45 when menstrual cycles become irregular, and falling to clearly below a thousand follicles at the time menstrual cycles cease, the event know as menopause [26-28]. In analogy to these histological changes, Scheffer *et al*. [11] demonstrated that the number of primordial follicles in the ovary, as published by Faddy and Gosden [29] correlated well with the number of growing follicles, counted by transvaginal sonography in the early follicular phase. So the decreasing size of the antral follicle cohort with age is a reflection of the decreasing primordial follicle pool. We used this principle to measure ovarian reserve, defined as the total number of follicles which can be stimulated under maximal ovarian stimulation with FSH. A number of the so-called ovarian reserve tests are supposed to indirectly reflect the size of the cohort of small antral follicles (2–10 mm in diameter) in the ovary. This decrease in follicle number is exemplified by the increased risk of producing a poor response in ovarian hyperstimulation in IVF patients at older age [30-32].

A gradual decrease with advancing age in the number of sonographically detectable antral follicles has been shown in many studies [3,25]. In recent years several papers have been published concerning the relation between the antral follicle count (AFC, defined as the total number of antral follicles, sized 2–5 or 2–10 mm, present in both ovaries) and the ovarian response in IVF [10,33], as well as the occurrence of the menopausal transition [34], indicating that this parameter relates strongly to the quantitative aspects of ovarian reserve.

The performance of AFC with regard to the prediction of poor response gave a sensitivity of 73% and a specificity of 95%, which would imply that the test performs only moderately, especially at the sensitivity level. In comparison with CCCT this sensitivity is lower whereas specificity seems the same. Consequently there would be more false negative patients with potential undertreatment as result. Increasing the threshold of AFC implies better sensitivity and to some extent, a still acceptable specificity. However the decrease of accuracy indicates that overall an unacceptable number of patients will be misdiagnosed.

Recently Hendriks *et al *[35], published a meta-analysis on the AFC as a predictor for poor ovarian response and concluded that AFC is an adequate test for the prediction of poor ovarian response, comparing to bFSH. The data of our study that does meet the criteria for inclusion in this meta analysis would fit seamlessly into the summary ROC curve of report. We confirm the previous observation of the little difference between CCCT and AFC [36].

The high intercycle stability of AFC [8] and its potentially likely attractive cost features, although formal cost effect comparison studies need to be done, are likely to make this test rather attractive for routine practice.

A great advantage of AFC over any other test is its potential usefulness for its ability to concomitantly predict low and high responders. So far EFORT seemed to have the best grades [14] but the current analysis provides evidence that AFC is superior. The test characteristics show us that an AFC > 14 could lead to the decision to adjust the gonadotrophin dose in trying to prevent a hyper response leading to OHSS. Of course the choice of the cut-off level depends on the appreciation of false positive and false negative results and on the consequences drawn by the clinician from an abnormal test.

Total volume of the ovaries detected by transvaginal ultrasound is correlated with the outcome parameters but not better than the count of antral follicles. Its performance was slightly to moderately less than that of AFC, both for poor and high response. Our data agree well with that published in a recent meta analysis [37].

In conclusion AFC performs well as a test for ovarian response being superior or at least similar to complex expensive and time consuming endocrine tests, probably most applicable in general practise.

Future studies will have to be carried out to determine if other ovarian reserve tests such as the measurement of Anti-Müllerian Hormone (AMH) [38-40] are better predictors for ovarian reserve.
